# Idiopathic pulmonary hemosiderosis and stroke secondary to protein C deficiency in a child with Down syndrome: a case report

**DOI:** 10.1186/s13256-023-03807-2

**Published:** 2023-03-11

**Authors:** Houda Ajmi, Chahra Bouafsoun, Nadia Arifa, Jalel Chemli, Saoussen Abroug

**Affiliations:** 1 Pediatrics Department, Sahloul University Hospital, 4054 Sousse, Tunisia; 2 Radiology Department, Sahloul University Hospital, 4054 Sousse, Tunisia

**Keywords:** Down syndrome, Hemosiderosis, Cerebral stroke, Protein C deficiency, Child

## Abstract

**Background:**

Patients with Down syndrome are at a higher risk of developing autoimmune disorders such as thyroiditis, diabetes, and celiac disease compared with the general population. Although some diseases are well known to be associated with Down syndrome, others such as idiopathic pulmonary hemosiderosis and ischemic stroke due to protein C deficiency remain rare.

**Case presentation:**

We report a case of a 2.5-year-old Tunisian girl with Down syndrome and hypothyroiditis admitted with dyspnea, anemia, and hemiplegia. Chest X-ray showed diffuse alveolar infiltrates. Laboratory tests showed severe anemia with hemoglobin of 4.2 g/dl without hemolysis. A diagnosis of idiopathic pulmonary hemosiderosis was confirmed by bronchoalveolar lavage showing numerous hemosiderin-laden macrophages, with a Golde score of 285 confirming the diagnosis of pulmonary hemosiderosis. Concerning hemiplegia, computed tomography showed multiple cerebral hypodensities suggestive of cerebral stroke. The etiology of these lesions was related to protein C deficiency.

**Conclusion:**

Idiopathic pulmonary hemosiderosis remains a severe disease, which is rarely associated with Down syndrome. The management of this disease in Down syndrome patients is difficult, especially when associated with an ischemic stroke secondary to protein C deficiency.

## Background

Patients with Down syndrome (DS) are usually followed for several health problems, especially immunological ones such as celiac disease, thyroiditis, diabetes, and so on. Although some of these diseases are well known to be associated with DS, others rarely occur in patients with DS. Few cases of idiopathic pulmonary hemosiderosis (IPH) have been reported in patients with DS. It is a rare and severe disease that predominantly affects children younger than 10 years of age [1]. IPH has an estimated incidence of 0.24–1.23 per million, with a mortality rate of 50% [2]. It is characterized by three signs: hemoptysis, sideropenic anemia, and alveolar and/or interstitial opacities on lung imaging. The diagnosis is confirmed by bronchoalveolar lavage (BAL) and/or lung biopsy [3], and patients with this disease may evolve pulmonary fibrosis [4]. The physiopathology of the association between DS and IPH remains unknown, and the management is difficult. In addition, the presence of chronic medical illness, frequently associated with DS, and the use of steroids may worsen the prognosis of this multimorbidity.

Ischemic stroke is a rare event in childhood. However, its occurrence is considerably more common in patients affected by DS [5]. Risk factors of stroke in DS have been mostly attributed to cardioembolic disorders [6]. Very few cases of children with DS affected by ischemic stroke secondary to protein C deficiency have been reported.

The association of IPH and DS has rarely been reported and the association between DS, stroke and IPH has, so far, never been reported.

We report the first case, to our knowledge, of a child with DS who presented with IPH and ischemic stroke secondary to protein C deficiency in the same onset.

## Case presentation

**Fig. 1:**
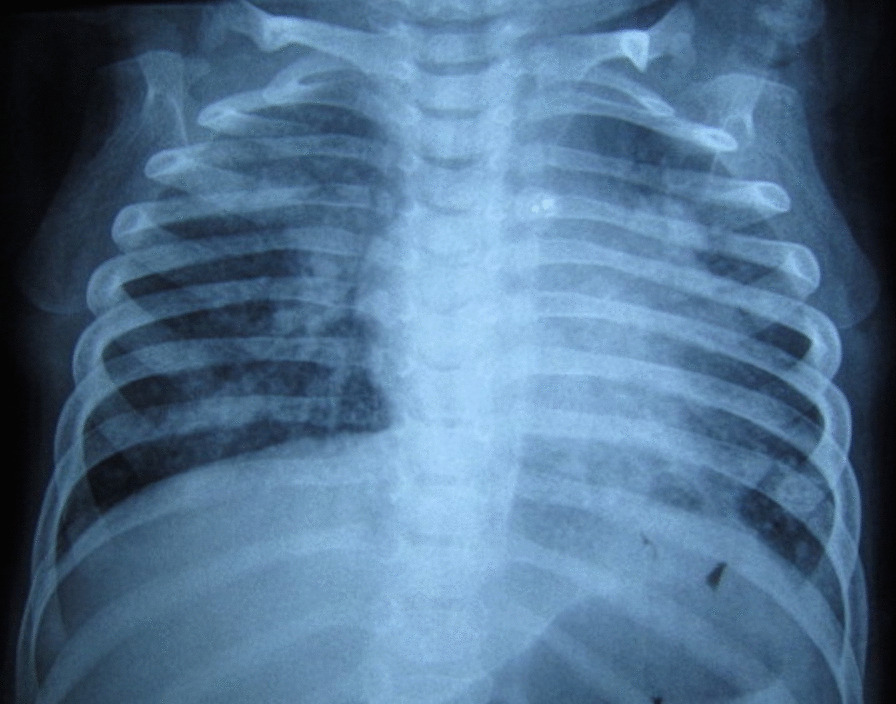
Chest X-ray showing diffuse alveolar infiltrates and no cardiomegaly

A 2.5-year-old Tunisian girl with DS, was admitted to our pediatric department with dyspnea, fever, and motor weakness. She was born at term to nonconsanguineous parents. During the first days of life, she was diagnosed with persistent ductus arteriosus. At the age of 12 months, the congenital heart defect was closed with catheterization. When she was 11 months, she was diagnosed with hypothyroidism secondary to Hashimoto’s thyroiditis and was treated with levothyroxine. Her parents reported recurrent lung infections and the occurrence of recurrent bronchopneumopathies, but no history of hematuria or thrombotic events. On admission, physical examination revealed a normal stature and microcephalic child with height of 83 cm [−1.8 standard deviation score (SDS)], weight of 11.1 kg (−1 SDS), and a head circumference of 45.5 cm (−2.1 SDS). She was pale, febrile (temperature of 38.2 °C), and had severe dyspnea. She had tachypnea at 60 breaths/minute with oxygen saturation in air at 70%. Her heart rate was at 140 beats/minute, her blood pressure was at 110/63 mmHg, and she had no heart murmur. Pulmonary auscultation found crackles and wheeze. Abdominal palpation showed splenomegaly and neurological examination showed isolated left hemiplegia. Chest X-ray showed diffuse alveolar infiltrates and no cardiomegaly (Fig. 1). The C-reactive protein was at 50 mg/l, and the complete blood count (CBC) identified severe anemia with hemoglobin of 4.2 g/dl, mean corpuscular volume of 70.1 μm^3^, mean corpuscular hemoglobin of 18.8 pg, white blood cells of 17,400/mm^3^, platelets of 169,000/mm^3^, and reticulocytosis of 223,400/mm^3^. The blood smear did not reveal schistocytes. Biochemical analysis showed no signs of hemolysis: total bilirubin of 10 μmol/l with a direct fraction of 2 μmol/l, haptoglobin of 2592.6 mg/l, and D-lactate dehydrogenase of 1295 UI/l. Blood urea nitrogen was 7.3 mmol/l, creatinine was 48 mmol/l, and serum ferritin was 71 ng/l. The level of transaminases was in the normal range (SGOT of 22 UI/l, SGPT of 39 UI/l). Microbiological analysis for bacteria, mycobacteria, and viruses, as well as visceral leishmaniasis serology and latex agglutination tests were negative. The direct antiglobulin test, electrophoresis of hemoglobin, and the erythrocyte fragility test showed no anomalies. Flexible bronchoscopy was performed. Bronchoalveolar lavage (BAL) showed 1,750,000 cells/ml (lymphocytes 6%; neutrophils 66%; macrophages 28%). Numerous hemosiderin-laden macrophages were detected (Golde score: 285) confirming the diagnosis of hemosiderosis. Bacteriological culture of the bronchoalveolar lavage fluid isolated neither bacteria isolates nor mycobacterium tuberculosis. Echocardiography showed no lesions and abdominal sonography identified an isolated splenomegaly. However, brain natriuretic peptide (BNP) was 3656 pg/ml. Immunoglobulin E was of 16 UI/ml. Immunoserological markers of celiac disease and autoimmune hepatitis, as well as antinuclear and antiphospholipid antibodies, were all negative. Laboratory investigations of macrophage activation syndrome (calcemia cholesterol, triglyceride, natremia, ferritin, and transaminases) were at normal ranges. Only the circulating anticoagulants were positive.

**Fig. 2:**
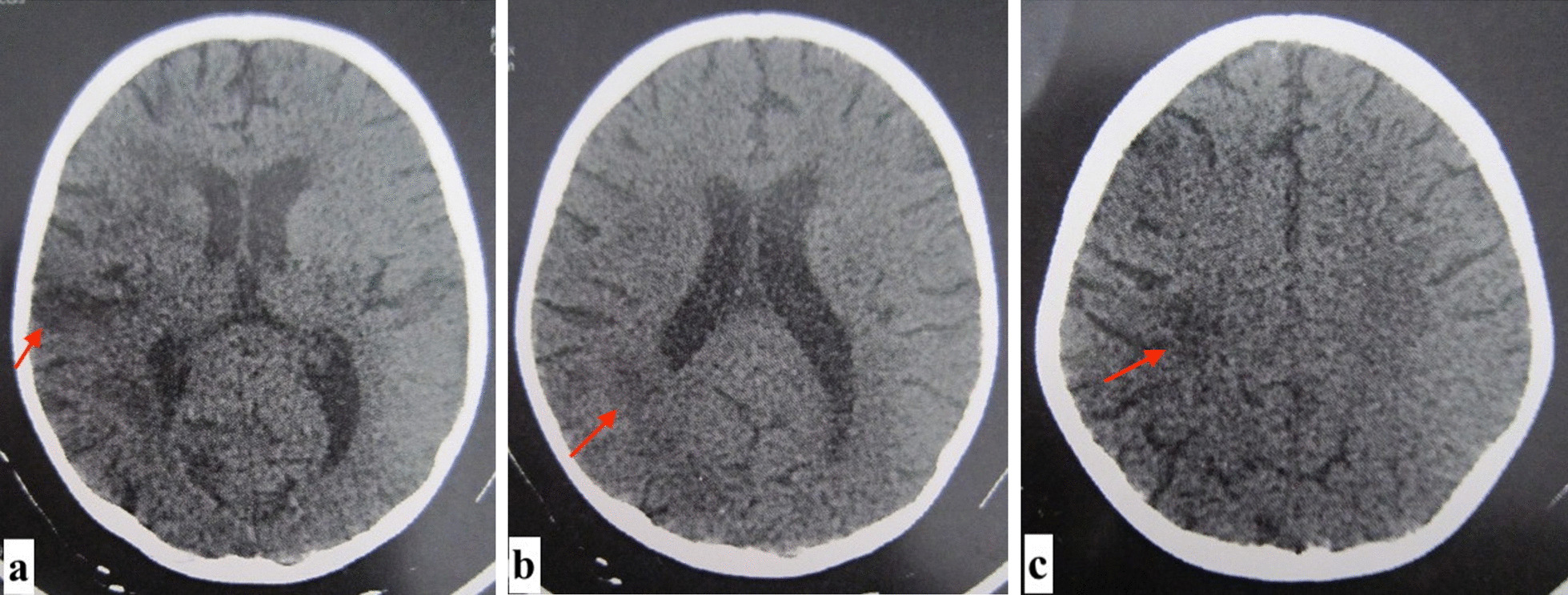
Non-compressive hypodensities in the right parietal cortex (arrows,** a**) and in the white matter areas (arrows** b**,** c**) related to the ischemic cerebral vascular accident

Concerning hemiplegia, brain computed tomography (CT) identified cortical and subcortical hypodensities located in the right parietal lobe, and hypodensities of the periventricular deep white substance and semi-oval center (Fig. 2). These lesions were suggestive of ischemic stroke. There was no intracerebral bleeding. Brain magnetic resonance imaging was not performed. Etiologic exploration of this ischemic stroke identified a protein C deficiency with protein C active or 28% (normal range 70–130%). This biological test was rechecked and the level of protein C was also decreased at 38%. Other biological investigations (hemoglobin electrophoresis, plasma homocysteine, protein S, antithrombin III, antinuclear and antiphospholipid antibodies) showed no anomalies. Repeated echocardiography also showed no anomalies. The diagnosis of idiopathic pulmonary hemosiderosis (IPH) associated with an ischemic stroke was held. The girl received a blood transfusion and was treated with prednisone (1 mg/kg/day) associated with inhaled corticosteroid (200 mcg) for her hemosiderosis. She was also given vitamin K antagonists for her protein C deficiency. However, she was hospitalized three times for relapse of this disease dyspnea because of poor treatment adherence. No immunosuppressive agent was included in the therapy since the efficacy of corticosteroid could not be verified.

After 1 year of evolution, the patient developed cervical lymphadenopathies with persistence of splenomegaly, and recurrences of dyspnea onsets and anemia. Thoraco–abdominal CT showed multiple cervical and thoraco–abdominal lymphadenopathies and splenomegaly. CT also identified a lung nodule in the right upper lobe.

Bone marrow examination showed no sign of malignancy and tuberculin skin test was negative. Histological analysis of one lymph node showed hyperplasia with evidence of epithelioid histiocytic granuloma and no areas of caseous necrosis. The etiological diagnosis of this lymphadenopathy was finally attributed to IPH. The patient was again put on corticosteroids (1 mg/kg/day) and inhaled corticosteroids (200 mcg/j) with no recurrence of his dyspnea and anemia during 1 year of follow-up.

## Conclusions

The association of IPH and DS has rarely been reported. The frequency of this association among children with IPH seems to be higher than that in the general population. Taytard *et al*. [7] reported a frequency of 20% of this association in a French pediatric cohort. Chin *et al*. [8] reported a frequency of 6.6%. The pathophysiology of IPH remains unknown. However genetic, allergic, environmental, and immunological factors seem to be involved in the development of the disease [7, 9]. Nevertheless, the autoimmune theory is recognized as the most acceptable theory, considering the frequent association with autoimmune diseases [4, 7] and the response to immunosuppressive therapy. Taytard *et al*. [7] reported 25 cases of children with IPH. The initial autoimmune screening of these patients revealed positive antineutrophilic cytoplasmic antibodies (40%), antinuclear antibodies (45%), and specific celiac disease antibodies (28%). In our case, the patient had positive antithyroid peroxidase and circulating anticoagulant antibodies. These immunological anomalies give evidence of the abnormal immunological features frequently described in DS. DS is well known to be associated with a significant increase of infectious, hematological, and autoimmune diseases, suggesting an intrinsic alteration of the immune system [10]. Several studies have reported disturbance of both cellular and humoral immunological response secondary to alterations of the expression of autoimmune regulator gene (located on chromosome 21), leading to thymic atrophy and functional impairments [10, 11]. These immune disturbances observed in DS result in a wide-ranging disease burden, such as IPH. Moreover, the majority of cases of patients having DS and IPH have been followed for at least one other autoimmune disease (Table 1). Clinical presentation of IPH includes dyspnea, hemoptysis, alveolar infiltrates on chest radiography, and iron deficiency anemia. Young children, like our patient, do not usually present with hemoptysis because they swallow their sputum. The diagnosis is confirmed by bronchoscopy with BAL fluid showing the presence of hemosiderin-laden macrophages (siderophages) or lung biopsy. The BAL fluid shows typically hemosiderin-laden macrophage ratio above 30% and/or a Golde score higher than 50 [12]. Based on the possible immunological pathogenesis of IPH, first-line conventional treatment of IPH usually includes systemic corticosteroids. Treatment with oral corticosteroids is used after the control of the acute phase of the disease. Although high-dose corticosteroids may reduce the morbidity and mortality of an acute episode, the effectiveness of long-term treatment remains controversial and exposes patients to the risk of serious side effects. Inhaled corticosteroids have been tried in some cases. A second-line immunosuppressive therapy including cyclophosphamide, azathioprine, hydroxychloroquine, or mycophenolate mofetil could be used as adjuncts to corticosteroids in children with dependence or resistance to corticosteroids. No immunosuppressive agent was prescribed to our patient. The efficacy of corticosteroid could not be verified in her because of her poor treatment adherence. Under treatment, patients should be evaluated by clinical assessment (hemoptysis, cough, dyspnea, full weight growth, clinical examination). Chest X-ray, CBC, and echocardiography are repeated every 3–6 months at the onset of the disease. Chest CT scan is done at the initial onset of the disease, then every 2–3 years as long as the disease seems to persist [13]. DS patients with IPH have a worse prognosis compared with other patients, including an increased mortality rate and frequent relapses [9]. Patients with DS progress early to pulmonary hypertension (PH) due to various factors including congenital heart disease, chronic airway obstruction, recurrent pulmonary infection, abnormal growth of the pulmonary vasculature, alveolar hypoventilation, and decreased number of alveoli [14, 15]. In our patient, the increased level of BNP could be a consequence of a possible PH, although the echocardiography did not shown tricuspid regurgitation to estimate the level of PH. Other than IPH, our patient presented simultaneously with cerebral stroke. This association in patients with DS has not been previously reported and the etiology is probably related to immunological disturbances associated with DS. The association of DS and protein C deficiency has been described in only one case by Gururaj *et al*. [16]. Genetic confirmation of this deficit was not done in that case nor in ours. In their case report, the authors explain that the rarity of this association may be due to the fact that protein C assay is not performed routinely in patients with DS presenting with stroke. On the other hand, it is possible that the decreased level of protein C is a secondary event to stroke.

**Table1:** Pulmonary hemosiderosis complicated by Down syndrome reported in the literature

References	Age	Sex	Medical history	Other associated diseases	Signs of onset	Diagnosis confirmation	Treatment	Prognosis
Aceti et al. [1]	4 years 7 months	Female	Recurrent respiratory infections	Ventricular and atrial septal defect, Patent ductus arteriosus	- Cough, Hemoptysis	Chest radiography CT scan BAL	Corticosteroids Hydroxychloroquine	Relapse occurring in the following year
Bakalli et al. [2]	13 years	Female	Severe anemia	Idiopathic thrombocytopenic purpura	Pallor, Extreme tiredness, Dyspnea	Chest X-ray CT scan Gastric lavage fluid	Corticosteroids Azathioprine	No relapses
Watanabe et al. [9]	9 years	Female	Fatigability Cough Vomiting of blood sputum	Hypothyroidism Autism	Hypoxemia Suspected acute pneumonia	Chest X-ray, CT scan Bronchoscopy	Oxygen supply Corticosteroid Antibiotic	Relapse 2 weeks after discharge treated by prednisolone with good clinical response
Alimi et al. [3]	2.9 ± 3.45 years	Four girls and five boys	**–**	PH (three patients) Congenital cardiopathy (four patients) Positive antinuclear antibodies (six cases)	Hemoptysis (two cases) Cough (two cases) Dyspnea (nine cases) Pneumonia (three cases)	Chest radiography CT scan BAL	Corticosteroids Hydroxychloroquine (one case) Immunosuppressive drugs (mycophenolate mofetil, cyclophosphamide, and/or azathioprime; three cases)	Relapses (six cases) PH (five cases) Death (three cases)

## Data Availability

The authors did not use any database, software, or tools for the writing of this manuscript.

## Abbreviations

BAL: Bronchoalveolar lavage
BNP: Brain natriuretic peptide
CBC: Complete blood count
CT: Computed tomography
DS: Down syndrome
IPH: Idiopathic pulmonary hemosiderosis
PH: Pulmonary hypertension
SDS: SD score
